# Reduced hippocampal and medial prefrontal gray matter mediate the association between reported childhood maltreatment and trait anxiety in adulthood and predict sensitivity to future life stress

**DOI:** 10.1186/2045-5380-4-12

**Published:** 2014-11-13

**Authors:** Adam X Gorka, Jamie L Hanson, Spenser R Radtke, Ahmad R Hariri

**Affiliations:** 1 Laboratory of NeuroGenetics, Department of Psychology and Neuroscience, Duke University, 417 Chapel Drive, Durham, NC 27708, USA; 2 Center for Developmental Science, University of North Carolina at Chapel Hill, 100 East Franklin Street, Suite 200 CB#8115, Chapel Hill, NC 27599, USA

**Keywords:** Stress, Hippocampus, Gray matter, Anxiety, MRI, Childhood maltreatment, Medial prefrontal cortex

## Abstract

**Background:**

The experience of early life stress is a consistently identified risk factor for the development of mood and anxiety disorders. Preclinical research employing animal models of early life stress has made inroads in understanding this association and suggests that the negative sequelae of early life stress may be mediated by developmental disruption of corticolimbic structures supporting stress responsiveness. Work in humans has corroborated this idea, as childhood adversity has been associated with alterations in gray matter volumes of the hippocampus, amygdala, and medial prefrontal cortex. Yet, missing from this body of research is a full understanding of how these neurobiological vulnerabilities may mechanistically contribute to the reported link between adverse childhood experiences and later affective psychopathology.

**Results:**

Analyses revealed that self-reported childhood maltreatment was associated with reduced gray matter volumes within the medial prefrontal cortex and left hippocampus. Furthermore, reduced left hippocampal and medial prefrontal gray matter volume mediated the relationship between childhood maltreatment and trait anxiety. Additionally, individual differences in corticolimbic gray matter volume within these same structures predicted the anxious symptoms as a function of life stress 1 year after initial assessment.

**Conclusions:**

Collectively, these findings provide novel evidence that reductions in corticolimbic gray matter, particularly within the hippocampus and medial prefrontal cortex, are associated with reported childhood maltreatment and individual differences in adult trait anxiety. Furthermore, our results suggest that these structural alterations contribute to increased affective sensitivity to stress later in life in those that have experienced early adversity. More broadly, the findings contribute to an emerging literature highlighting the critical importance of early stress on the development of corticolimbic structures supporting adaptive functioning later in life.

## Background

Stress early in life is associated with increased rates of mood and anxiety disorders in adulthood [1] with a recent meta-analysis showing a 62% increase in the risk for anxiety disorders in individuals who have suffered early trauma [2]. With over one in eight children in the US experiencing early adversity such as child maltreatment [3], this represents a major public health problem. Though well-studied and well-replicated in psychological and epidemiological research, little is known regarding the mechanisms mediating the association between adverse childhood experiences and later affective psychopathology, particularly anxiety disorders.

Preclinical research has begun to uncover potential neurobiological mechanisms underlying these relationships, as a multitude of animal models of early life stress (e.g., maternal deprivation, maternal abuse), result in an increase in anxiety-like behavioral phenotypes and also structural alterations in corticolimbic regions such as the hippocampus, amygdala, and prefrontal cortex [4,5]. Similar alterations have been noted in human samples exposed to childhood maltreatment, with reduced gray matter volume within the hippocampus and alterations in an interconnected network of corticolimbic structures, including the amygdala, orbitofrontal cortex, and anterior cingulate cortex as a function of stressors experienced during childhood [6-10]. Such results have important implications for the vulnerability to affective psychopathology. Reduced gray matter within these brain structures has also been associated with higher trait anxiety as well as mood and anxiety disorders in adults [11-17].

Missing from this body of research, however, are strong links to behavior, as many of the past studies have simply noted brain differences between groups. Rao and colleagues [18] took an important initial step to close this gap, finding decreased hippocampal gray matter mediated the relationship between adversity during childhood and increased risk for major depression. While these investigators importantly connected alterations in neurobiology and behavior, it is still unclear how differences in the brain are associated with higher rates of affective dysregulation after early stress. Further studies are needed to drill down to understand how neurobiological vulnerabilities may mechanistically contribute to the reported link between adverse childhood experiences and later affective psychopathology.

Related to this idea, a growing body of psychological research has found that environmental stressors often play a precipitating role in the onset of mood and anxiety disorders and that the experience of stress early in life confers risk for psychopathology by sensitizing organisms to stress in adulthood [19,20]. Tellingly, childhood maltreatment is further associated with increased trait anxiety, alterations in hypothalamic-pituitary-adrenal (HPA) axis stress reactivity, and greater potentiated startle to threat [1,21,22]. These collective findings suggest that early adversity may create a dispositional sensitivity to perceived threat and may be associated with elevated risk for mood and anxiety disorders following stressful life events in adulthood [23-25]. No study to date, however, has formally linked individual differences in the intermediate risk phenotype of trait anxiety with the experience of childhood maltreatment, particularly in relation to later stress, and associated changes in corticolimbic morphology. It is likely that alterations in corticolimbic gray matter associated with childhood maltreatment interact with experiences of stress in the future and these neural phenotypes associated with early adversity may aid researchers in prospectively predicting relative risk and resilience in the context of environmental challenge.

In the current study, we examined if the association between self-reported childhood maltreatment and the expression of trait anxiety in adulthood is mediated by differences in corticolimbic morphology. Specifically, we tested whether childhood maltreatment was associated with trait anxiety via reductions in gray matter within neural circuits regulating stress responsiveness. To this end, structural MRI and self-reported measures of childhood maltreatment, recent life stress, and negative affect were examined in 818 participants of the ongoing Duke Neurogenetics Study (DNS). A subset of 196 participants completed additional behavioral assessments at least 1 year after the DNS. This unique longitudinal component allowed us to further determine if the structural correlates of childhood maltreatment predict subsequent vulnerability to future stressful life events. Based on prior preclinical and clinical research, we hypothesized that childhood maltreatment would be negatively correlated with corticolimbic gray matter volume with a specific focus on the hippocampus, amygdala, and medial prefrontal cortex (mPFC) including the orbitofrontal cortex (OFC) and anterior cingulate cortex (ACC). Further, we hypothesized that gray matter reductions within these structures would mediate the relationship between childhood maltreatment and adult trait anxiety. Given the importance of these corticolimbic structures in orchestrating adaptive responses to stress, we lastly hypothesized that these gray matter reductions would predict increased anxiety, following stressful life events in the future.

## Methods

### Participants

This study was approved by the Duke University Medical Center Institutional Review Board. Data were available from 906 participants who had successfully completed the ongoing DNS, which assesses a wide range of behavioral and biological traits among non-patient, 18–22-year-old university students. All participants provided informed consent in accordance with Duke University Medical Center Institutional Review Board guidelines prior to participation. The participants were in good general health and free of the following study exclusions: (1) medical diagnoses of cancer, stroke, head injury with loss of consciousness, untreated migraine headaches, diabetes requiring insulin treatment, chronic kidney or liver disease, or lifetime history of psychotic symptoms; (2) use of psychotropic, glucocorticoid, or hypolipidemic medication; and (3) conditions affecting cerebral blood flow and metabolism (e.g., hypertension). As diagnoses of mood disorders are associated with gray matter volume and trait anxiety, we excluded participants based on a diagnosis of any past or current DSM-IV axis I or select axis II (antisocial and borderline personality) disorders as identified through clinical interviews using the electronic MINI [26]. The participants were administered a neuropsychological battery that included the Wechsler Abbreviated Scale of Intelligence (WASI) [27].

Analyses testing primary hypotheses were limited to 818 participants (469 females, mean age =19.62 ± 1.24 SD) with overlapping structural MRI and self-report data surviving our stringent, multilevel quality control procedures described below. All successful DNS participants are contacted every 3 months after initial study completion and asked to complete a brief online assessment of recent life events, mood, and affect in the past week. Secondary longitudinal analyses were restricted to a subset of 196 participants (123 females, mean age =19.49 ± 1.17 SD) that completed these follow-up measures at least 1 year after their structural scan (mean time since scan = 466.98 days ±131.66 at the time of follow-up). See Table 1 for descriptive statistics of the full sample and longitudinal subsample.

**Table 1:** Descriptive statistics of all participants

	**Age**	**Gender**	**Childhood maltreatment (CTQ)**	**Recent life stress (LESS)**	**Trait anxiety (STAI-T)**	**IQ score**^**a **^**(WASI)**	**State anxiety (MASQ-AA)**	**Days since MRI scan**
Full sample (*N* =818)	19.6 ± 1.24	349 male, 469 female	33.2 ± 8.12	4.2 ± 2.95	37.2 ± 5.65	121.62 ± 8.57		
Longitudinal sample (*N* =196)	19.5 ± 1.17	73 male, 123 female	32.5 ± 7.26	2.2 ± 2.37	37.0 ± 9.0		20.9 ± 5.75	477.0 ± 131.66

Full sample: all participants who completed the structural MRI, trait anxiety assessment (STAI-T), and inventories of life stress (CTQ, LESS) and were free of current or past mood and anxiety disorders. Longitudinal sample: subset of those participants who completed a follow-up assessment at least 1 year after their MRI scan.

^a^WASI IQ scores were not collected for one participants (*N* =817).

### Self-report measures

The State-Trait Anxiety Inventory - Trait (STAI-T) version was used to assess the general tendency with which individuals perceive encountered situations to be threatening and to respond to such situations with subjective feelings of apprehension and tension [28].

The Childhood Trauma Questionnaire (CTQ) was used to assess exposure to childhood maltreatment in five categories: emotional, physical and sexual abuse, and emotional and physical neglect [29]. Each of the instrument’s subscales has robust internal consistency and convergent validity with a clinician-rated interview of childhood abuse and therapists’ ratings of abuse [30]. As each type of stressor would contribute to allostatic load which is thought to impact the hippocampus, we conducted analyses using total scores, summed from all subscales, which have previously been demonstrated to predict gray matter reductions within the brain [6,31].

We used a modified version of the Life Events Scale for Students (LESS) [32] to assess the occurrence and self-reported subjective impact of common stressful life events within the past 12 months (see Additional file 1 for details on items). As the experience of each life event would contribute to allostatic load, which is thought to impact corticolimbic circuitry, we conducted analyses using cumulative impact scores (i.e., the sum of self-reported impact from all stressful life events reported within the last 12 months).

Recent negative affect was assessed using the Mood and Anxiety Symptom Questionnaire (MASQ) [33]. The MASQ is a well-validated measure yielding four subscales assessing symptoms experienced within the last 7 days specific to Anxious Arousal (MASQ-AA) or Anhedonic Depression (MASQ-AD) as well as General Distress Anxiety (MASQ-GDA) and General Distress Depression (MASQ-GDD). Previous research has demonstrated that MASQ-AA, which represents items assessing physiological arousal, has higher discriminant validity than MASQ-GDA, which represents items assessing general and nonspecific distress [33,34]. Here, we utilize MASQ-AA as it best represents a state measure related to our measure of trait anxiety.

### Acquisition of structural MRI data

Each participant was scanned on one of two identical research-dedicated GE MR750 3T scanners at the Duke-UNC Brain Imaging and Analysis Center. Each identical scanner was equipped with high-power high-duty cycle 50-mT/m gradients at 200 T/m/s slew rate and an eight-channel head coil for parallel imaging at high bandwidth up to 1 MHz. For optimized voxel-based morphometry (VBM), T1-weighted images were obtained using a 3D Ax FSPGR BRAVO sequence with the following parameters: TR =8.148 s; TE =3.22 ms; 162 sagittal slices; flip angle, 12°; FOV, 240 mm; matrix =256 × 256; slice thickness =1 mm with no gap; and total scan time =4 min and 13 s.

### Optimized voxel-based morphometry

Regional gray matter volumes from T1-weighted images were determined using the VBM8 toolbox (version 369 http://dbm.neuro.uni-jena.de/vbm/) within SPM8. The toolbox is an extension of the unified segmentation model [35]. Using this approach, individual T1-weighted images were segmented into gray, white, and CSF images using an adaptive maximum a posterior technique, partial volume estimation, an optimized block-wise nonlocal means denoising filter and spatial constraints based on a classical Markov random field model. Resulting gray matter images were normalized to a gray matter template in Montreal Neurological Institute (MNI) space using affine transformations. Subsequently, gray matter voxel values were scaled using the Jacobian matrix parameters from normalization to adjust for volume changes during the affine transformation. In line with the methodology of Good et al. [36], normalized gray matter images were then smoothed with a 12-mm FWHM kernel [36].

### Hypothesis testing

Associations between CTQ total scores and local gray matter volume were assessed by entering the resulting processed whole brain gray matter images into a second-level multiple regression analysis within SPM8. To ensure that results were unique to CTQ scores, we controlled for recent life stress (LESS cumulative impact) in addition to age and gender. We corrected against type I error in these analyses resulting from multiple comparisons by applying a *p* <0.05 family wise error (FWE)-corrected threshold on a voxel level across bilateral medial temporal lobe and medial PFC regions of interest, which were defined using a medial frontal gyrus regions of interest (ROI) selected from the Talairach Daemon - Labels toolbox and a medial temporal lobe ROI, comprised of bilateral hippocampus and amygdala masks, using the Automatic Anatomical Labeling (AAL) toolbox within SPM8. Gray matter volumes from a 5-mm sphere surrounding the max voxel of resulting clusters showing significant associations with CTQ scores were then extracted and entered into SPSS v21 for mediation and moderation analyses using the PROCESS macro [37].

Our mediation analyses assessed the indirect effect (the *a* path multiplied by the *b* path) of self-reported childhood maltreatment on trait anxiety via variability in gray matter volume. Previous research has demonstrated that the indirect effect, in contrast with the variables used to calculate it, is only normally distributed in special cases which can lead to unbalanced confidence limits [38]. Consequently, we determined significance using confidence intervals obtained using bias-corrected bootstrapping which previous research suggests is the best resampling method for testing indirect effects [38]. Reported regression coefficients reflect standardized betas.

## Results

### Demographic data

The observed means and distributions of our self-report measures were broadly in line with previous reports. For the CTQ total scores, the mean was 33.03 for men and 33.33 for women with a range of 25–75 (score of 25 was the minimum possible value with participants selecting a “1 = Never True” for all items). Previous CTQ total scores derived from young adults in a community sample was 32.96 for men and 30.27 for women [39]. Although ranges were not reported for this community sample, the 90th percentile had a mean of 41.00 for men and 41.49 for women, while the 90th percentile in our sample has a mean of 49.15 for men and 52.46 for women. Previous assessments of cumulative impact for all 36 LESS items in undergraduate samples report means of 248.32 with a range of 0–1,009 (with impact for each event scored on a scale of 0–100) [40]. The version of the LESS collected in this study assessed 45 items (see Additional file 1 for all items) with impact scored on a scale of 1–4 for each event. Although we collect data on a broader number of negative life events, using a truncated Likert scale for impact rating, our results (mean =9.26, range: 0–52) are broadly in line with previous reports after scaling adjustments (mean =231.57, range: 0–1,300). STAI-T scores from our sample (mean =37.21, range: 20–71) are similar to those reported from other samples of young adults (mean =32.68 for men and 36.85 for women) [41].

Our MASQ-AA scores (mean =20.9, range: 17–52) included values that were larger than those observed in previous reports from undergraduate samples (mean =18.63, range: 17–23; and mean =18.70, range: 17–26) [42] (see Table 1 for additional demographic information).

### Primary analyses (full sample)

#### Childhood maltreatment and corticolimbic gray matter volume

**Figure 1:**
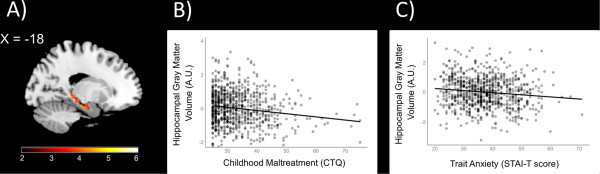
**Relationships between hippocampal gray matter volume, childhood maltreatment, and trait anxiety (*N *= 818). (A)** Statistical parametric map from medial temporal lobe ROI analyses illustrating voxels exhibiting a significant negative correlation with CTQ total scores within the left hippocampus while controlling for age, gender, and recent life stress (LESS) (*x* = −18). **(B)** Scatterplot depicting hippocampal gray matter volume from a 5-mm sphere surrounding max voxel (*x* = −18, *y* = −21, *z* = −18) as a function of CTQ total scores. **(C)** Trait anxiety plotted against gray matter volumes from 5-mm sphere. Scatterplots reflect partial correlations between variables after controlling for gender, age, and recent life stress (LESS). *Y* axes reflect standardized residuals. (AU = arbitrary units).

**Figure 2:**
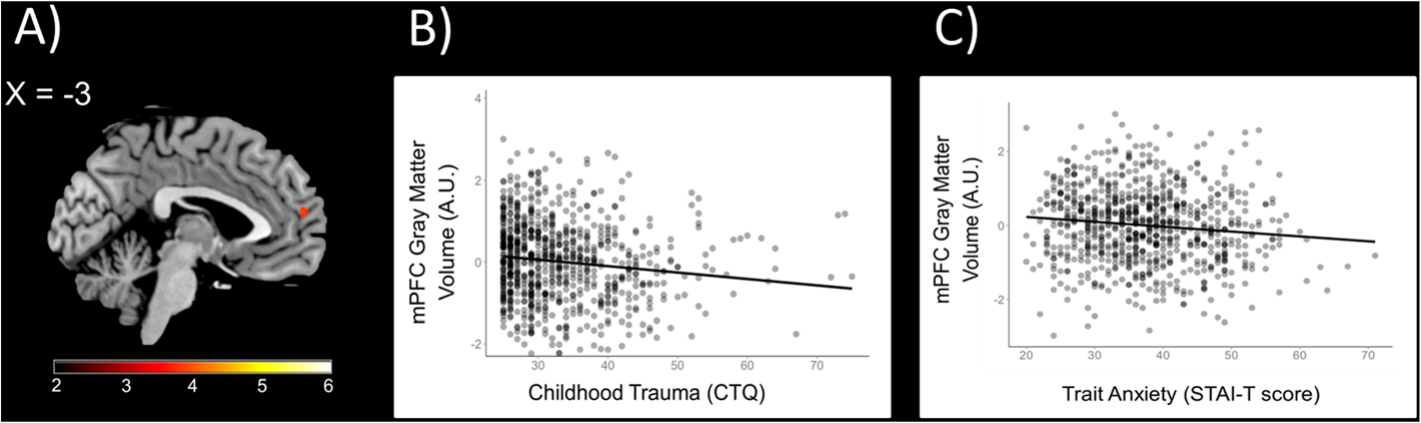
**Relationships between mPFC gray matter volume, childhood maltreatment, and trait anxiety (*N *= 818). (A)** Statistical parametric map from frontal gyrus ROI analyses illustrating voxels exhibiting a significant negative correlation with CTQ total scores within the mPFC while controlling for age, gender, and recent life stress (LESS) (*x* = −3). **(B)** Scatterplot depicting mPFC gray matter volume from a 5-mm sphere surrounding max voxel (*x* = −3, *y* =57, *z* =16) as a function of CTQ total scores. **(C)** Trait anxiety plotted against mPFC gray matter volumes from 5-mm sphere. Scatterplots reflect partial correlations between variables after controlling for gender, age, and recent life stress (LESS). *Y* axes reflect standardized residuals. (AU = arbitrary units).

CTQ total scores were negatively correlated with gray matter volume within the left hippocampus after controlling for recent life stress cumulative impact scores, gender, and age (*x* = −18, *y* = −21, *z* = −18; *T* =4.46; *p* <0.05, FWE-corrected; 334 voxels; Figure 1A). No significant correlations were observed between CTQ total scores and gray matter volume within the right hippocampus or bilateral amygdala. CTQ total scores were, however, negatively correlated with gray matter volume within the mPFC (*x* = −3, *y* =57, *z* =16; *T* =3.88; *p* <0.05, FWE-corrected; 19 voxels; Figure 2A).

### Correlations with trait anxiety

**Figure 3:**
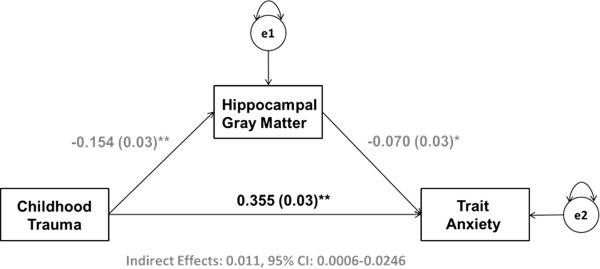
**Hippocampal gray matter volume mediates the relationship between childhood maltreatment and trait anxiety in adulthood.** Results from a path analysis testing a model wherein left hippocampal gray matter volume mediates the relationship between childhood maltreatment and trait anxiety in adulthood. Values represent standardized parameter estimates with standard errors presented in parentheses after controlling for age, gender, and recent life stress (LESS). The circles labeled e1 and e2 denote the variance in left hippocampal gray matter volume and trait anxiety scores unaccounted for by the model. *p <0.05, **p <0.005.

**Figure 4:**
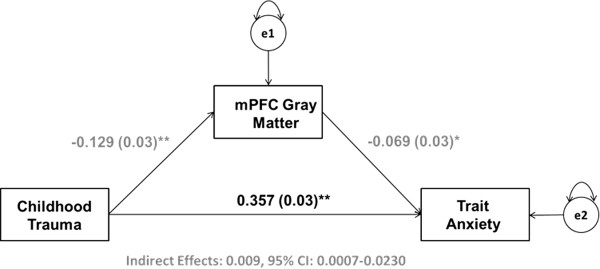
**mPFC gray matter volume mediates the relationship between childhood maltreatment and trait anxiety in adulthood.** Results from a path analysis testing a model wherein mPFC gray matter volume mediates the relationship between childhood maltreatment and trait anxiety in adulthood. Values represent standardized parameter estimates with standard errors presented in parentheses after controlling for age, gender, and recent life stress (LESS). The circles labeled e1 and e2 denote the variance in mPFC gray matter volume and trait anxiety scores unaccounted for by the model. *p <0.05, **p <0.005.

CTQ total scores were positively correlated with trait anxiety as assessed by the STAI-T (*β* =0.366, *p* <0.001). Individual differences in gray matter volumes from 5-mm spheres surrounding the max voxels within the left hippocampus and mPFC identified from the regression analyses with CTQ total scores reported above were correlated with trait anxiety (left hippocampus: *β* = −0.126, *p* <0.001; mPFC: *β* = −0.116, *p* <0.001; Figures 1C and 2C). Mediation analyses revealed that left hippocampal gray matter volume significantly mediated the relationship between CTQ total scores and STAI-T scores (*β* =0.011, lower limit confidence interval (LLCI) =0.0006, upper limit confidence interval (ULCI) =0.0246; Figure 3). Mediation analyses further demonstrated that mPFC gray matter volume significantly mediates the relationship between CTQ total scores and STAI-T scores (*β* =0.009, LLCI =0.0007, ULCI =0.0230; Figure 4). These significant relationships were all robust to inclusion of LESS cumulative impact scores, gender, and age as covariates.

### Secondary analyses (longitudinal sample)

#### Corticolimbic morphology and responsiveness to stress

**Figure 5:**
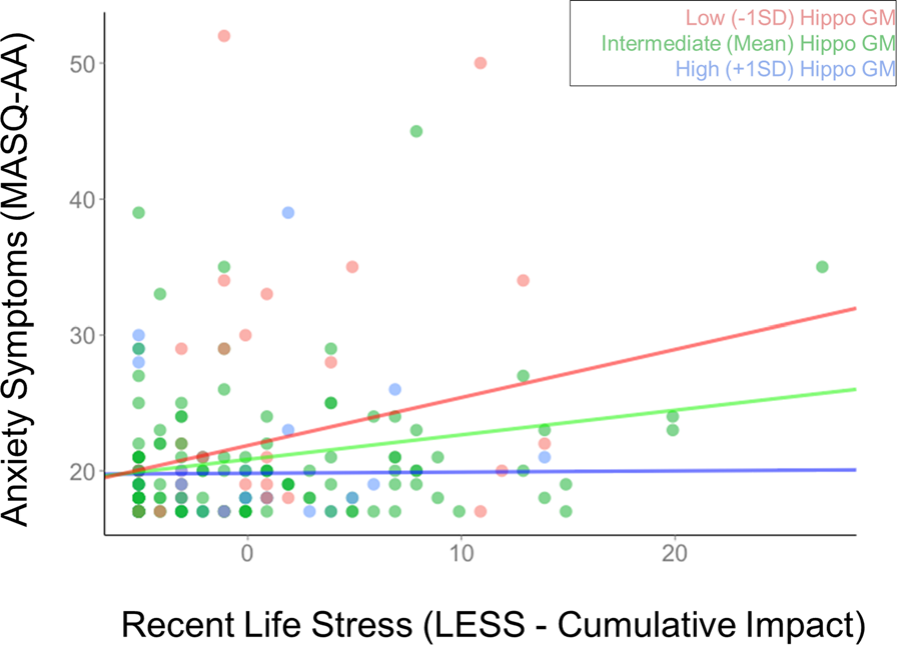
**The relationship between recent life stress (LESS) and current anxiety symptoms (MASQ-AA).** The relationship between recent life stress (LESS) and current anxiety symptoms (MASQ-AA) measured at least 1 year after initial assessment, plotted at low (−1 SD), intermediate (mean), and high (+1 SD) levels of left hippocampal gray matter volume. Scatterplots reflect partial correlations between LESS and STAI-T after controlling for gender, age, and time since MRI scan. MASQ-AA scores reflect standardized residuals.

**Figure 6:**
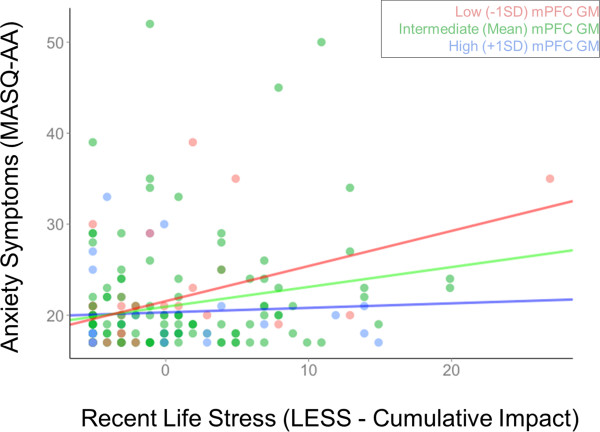
**The relationship between recent life stress (LESS) and current anxiety symptoms (MASQ-AA).** The relationship between recent life stress (LESS) and current anxiety symptoms (MASQ-AA) measured at least 1 year after initial assessment, plotted at low (−1 SD), intermediate (mean), and high (+1 SD) levels of mPFC gray matter volume. Scatterplots reflect partial correlations between LESS and STAI-T after controlling for gender, age, and time since MRI scan. MASQ-AA scores reflect standardized residuals.

There was a significant positive correlation between LESS cumulative impact scores in the year following initial assessment and MASQ-AA scores at follow-up (*β* =0.204, *p* =0.008) after controlling for age, gender, and number of days between MRI scan and follow-up assessment. This correlation was significantly moderated by the same left hippocampal gray matter volumes described above (*β* = −0.181, *p* =0.038) (Figure 5) while controlling for covariates. The participants with relatively less hippocampal gray matter volume exhibited stronger correlations between LESS and MASQ-AA than those with average or relatively more gray matter volumes (simple slopes: −1 SD: *β* =0.345, *p* =0.001; mean: *β* =0.164, *p* =0.033, +1 SD: *β* = −0.017, *p* =0.893; Figure 4). Gray matter within the medial prefrontal cortex similarly moderated the relationship between LESS cumulative impact scores and MASQ-AA (interaction term: *β* = −0.178, *p* =0.008; simple slopes: −1 SD: *β* =0.378, *p* =0.002; mean: *β* =0.200, *p* =0.008, +1 SD: *β* =0.023, *p* =0.823; Figure 6) while controlling for covariates.

## Discussion

Here, fitting with past research, we find that self-reported childhood maltreatment was associated with reduced gray matter volumes within the medial prefrontal cortex and left hippocampus. We also for the first time to date, formally demonstrate that gray matter volume reductions within the hippocampus and mPFC mediate the association between the self-reported childhood maltreatment and the increased expression of trait anxiety in adulthood. Our results expand on prior preclinical and clinical research and begin to fill in important gaps in the understanding of the sequelae of early life stress including associated variance in neurobiology. Unique to our work, we then attempted to link these neurobiological phenotypes to susceptibility to environmental challenge in the future by focusing on the relationship between stress later in life and state anxiety as a function of individual differences in corticolimbic morphology. Consistent with our hypotheses, our secondary analyses demonstrate this intermediate behavioral phenotype is related to reduced hippocampal and mPFC gray matter volume. As such, gray matter within these structures may represent a neural embedding of early life stress through which later psychopathology emerges.

Our findings of structural alterations within the hippocampus and mPFC associated with self-reported childhood maltreatment are broadly consistent with findings from animal models showing decreased dendritic arborization, spine density, and neuronal number within these areas [43-45]. Though, care should be taken in direct translation, as the resolution of MRI precludes focusing on neural architecture at that level and our data is correlative in nature. Thinking about what these neural alterations may mean for behavior, the hippocampus is critical in shaping emotional responses to environmental challenge through its regulation of the HPA axis and encoding of contextual memory for emotional experiences as exemplified by fear learning [40,41]. The mPFC is essential for the process of fear extinction and functions to regulate behavioral and neuroendocrine responses to controllable stressors [46,47]. The hippocampus and mPFC both support multiple processes that likely contribute to trait anxiety; however, it is currently unclear which exact processes are being affected by gray matter reductions in these regions.

Of note, our study contained both (primary) cross-sectional and (secondary) longitudinal assessments. The deployment of longitudinal designs is especially important when considering how stress affects brain structure across development and how early adversity shapes future responses to stress. Although much research has addressed the relationship between childhood maltreatment and gray matter volume in adulthood, certain studies provide unique insight into the complexity of these relationships. Meta-analyses demonstrate that childhood maltreatment is associated with reduced hippocampal gray matter in adulthood, but not during childhood [34] suggesting that the impact of adversity on neural morphology is not immediate but develops over time. A better understanding of how childhood maltreatment is associated with gray matter in neural structures that generate and regulate responses to stress may facilitate our understanding of how early adversity impacts risk in the context of stress over the lifespan and may implicate specific developmental windows during which treatment and prevention strategies are most effective. It is possible that intervention strategies immediately following childhood maltreatment may prevent the emergence of hippocampal deficits, which are associated with risk for mood and anxiety disorders. Preventing these morphological changes via intervention during childhood may be more effective than treating the negative sequelae that emerge in adulthood.

Our work is not without limitations. First, our measures of self-reported childhood maltreatment, trait anxiety, and gray matter morphology were assessed concurrently, and as such are correlative in nature and cannot establish temporal order. For example, it is possible that persons who are high in trait anxiety, in addition to having reduced gray matter volume, are also more likely to retrospectively remember or report maltreatment during childhood. It is thus possible that hippocampal and mPFC gray matter volume mediates the impact of trait anxiety on self-reported childhood maltreatment, rather than vice versa. However, our finding that individual differences in gray matter volume within these structures prospectively predicts anxious arousal and subsequent to the experience of stressful life events supports the importance of gray matter deficits within the hippocampus and mPFC associated with childhood maltreatment as a mechanism through which sensitivity to stressful life events emerges. Developmental longitudinal studies in high-risk populations (e.g., individuals with positive family history for disorder) could advance the relevance of this potential mechanism for understanding etiology and pathophysiology of anxiety disorders.

Additionally, we observed decreased gray matter within the left hippocampus as a function of self-reported childhood maltreatment, but no effect of early life stress was significant within the right hemisphere. We did not hypothesize a lateralized effect, and it is possible that results in the right hemisphere are simply less robust. A recent meta-analysis found evidence for bilateral reductions in hippocampal gray matter in participants with PTSD related to childhood trauma [34]. We observe only a weak effect of childhood maltreatment on gray matter volume within the right hippocampus and only at more liberal statistical threshold (*p* <0.005 uncorrected, 21 voxels). Future research will be needed to determine whether gray matter reductions in the right hippocampus are linked to vulnerability to stress similarly to the results reported here.

Lastly, the effect sizes observed in our analyses are relatively small. Further, our sample consists of undergraduate students who report experiencing childhood maltreatment yet are free of past or current mood and anxiety disorders. As such, this sample may represent a relatively resilient population, and it is not immediately clear if our results have direct parallels to clinical outcomes. Nonetheless, previous research has demonstrated that reduced gray matter within the hippocampus mediates the association between early life adversity and vulnerability to major depression [42], which parallels the results reported here. Additionally, the age of our participants (18–22 years of age) is relatively young compared to the average onset of mood and anxiety disorders [48,49], and it is possible that some of our participants will go on to develop psychopathology within their lifetime. Future research will be needed to determine whether gray matter reductions within healthy participants exist on a continuum with clinical samples, and whether gray matter morphology is associated with responses to stress later in life in a manner that is clinically meaningful.

These limitations notwithstanding, our results suggest that structural variance in hippocampal and mPFC gray matter volume represent mechanisms through which childhood maltreatment may shape not only the expression of trait anxiety but also the responsiveness to stress. By specifically modeling the effects of childhood maltreatment onto behavioral processes indirectly through variability in neural phenotypes, our work can serve as a springboard for future research. A wealth of preclinical and clinical evidence suggests that the experience of stress early in life and higher levels of trait anxiety are risk factors for the development of mood and anxiety disorders. Our results suggest that structural changes within the hippocampus and mPFC may represent the neural embedding of early life stress, which shapes risk for subsequent psychopathology by affecting how we respond to challenges in the environment.

## Conclusions

Our findings suggest that reduced corticolimbic gray matter, particularly within the hippocampus and medial prefrontal cortex, mediates the relationship between reported childhood maltreatment and individual differences in adult trait anxiety. Further, our results suggest that these structural alterations contribute to increased affective sensitivity to stress later in life. These findings contribute to the literature addressing how early stress may affect the development of corticolimbic structures supporting adaptive functioning later in life.

## Abbreviations

AAL: Automatic anatomical labeling; ACC: Anterior cingulate cortex; AU: Arbitrary units; CTQ: Childhood trauma questionnaire; DNS: Duke neurogenetics study; FWE: Family wise error; HPA: Hypothalamic-pituitary-adrenal; LESS: Life events scale for students; LLCI: Lower limit confidence interval; MASQ-AA: Mood and anxiety symptom questionnaire - anxious arousal; MASQ-GDA: Mood and anxiety symptom questionnaire - general distress anxiety; MNI: Montreal neurological institute; mPFC: Medial prefrontal cortex; OFC: Orbitofrontal cortex; ROI: Region of interest; STAI-T: State-trait anxiety inventory - trait version; ULCI: Upper limit confidence interval; VBM: Voxel-based morphometry; WASI: Wechsler abbreviated scale of intelligence.

## Competing interests

The authors declare that they have no competing interests.

## Authors’ contributions

AXG performed the statistical analyses, participated in the study design and data collection, and helped draft the manuscript. JLH helped draft the manuscript and aided with the statistical analyses. SRR helped draft the manuscript and participated in the data collection. ARH conceived of the study, participated in the study design, and helped draft the manuscript. All authors read and approved the final manuscript.

## Supplementary Material

Additional file 1 **Modified Life Events Scale for Students (LESS).** This supplementary file contains the items used in our modified version of the Life Events Scale for Students. Click here for file
