# Supplementary material for: Reduced hippocampal and medial prefrontal gray matter mediate the association between reported childhood maltreatment and trait anxiety in adulthood and predict sensitivity to future life stress
Source: Biol Mood Anxiety Disord. 2014 Nov 13;4:12. doi: 10.1186/2045-5380-4-12 (PMC4236295; doi:10.1186/2045-5380-4-12)
Supplement: Additional file 1 — Modified Life Events Scale for Students (LESS). This supplementary file contains the items used in our modified version of the Life Events Scale for Students. [file 2045-5380-4-12-S1.docx]

**Supplementary Material**

**Modified Life Events Scale for Students (LESS)**

The responses to the occurrence in the last year of the following LESS items were used in analyses:

1 Death of a parent

2 Major personal injury or illness

3 Major argument with parents

4 Beginning an undergraduate program at university

5 Moving away from home

6 Failing a number of courses

7 Minor violation of the law (e.g., speeding ticket)

8 Getting kicked out of college

9 Pregnancy (either yourself or being the father)

10 Minor car accident

11 Major violation of the law (e.g., sentenced with jail term (self))

12 Moving out of town with parents

13 Spouse or boy/girlfriend died

14 Establishing new steady relationship with partner

15 Finding a part‐time job

16 Sex difficulties with boy/girlfriend

17 Failing a course

18 Major change of health in close family member

19 Major car accident (car wrecked, people injured)

20 Death of your best or very close friend

21 Serious illness of your best or very close friend

22 Serious personal crisis experienced by family member or very good friend

23 Major housing problems

24 Breaking up of parent's marriage/divorce

25 Losing a part‐time or a full‐time job

26 Major and/or chronic financial problems

27 Major argument with boy/girlfriend or spouse

28 Parent losing a job

29 Switch in program within same college or university

30 Losing a good friend

31 Change of job

32 Break‐up with boy/girlfriend or spouse

33 Minor financial problems

34 Major conflict with a family member or very close friend

35 Assault, rape, or mugging

36 Major difficulties at work

37 Lost driver's license

38 Pet died

39 Was robbed

40 Infidelity on the behalf of the spouse / significant other

41 Birth of a child

42 Abortion

43 Miscarriage

44 Found out that cannot have children

45 Child died
